# *APOE* ε4 is associated with decreased synaptic density in cognitively impaired participants

**DOI:** 10.1002/alz.13775

**Published:** 2024-03-13

**Authors:** Kun He, Binyin Li, Jie Wang, Ying Wang, Zhiwen You, Xing Chen, Haijuan Chen, Junpeng Li, Qi Huang, Qihao Guo, Yiyun Henry Huang, Yihui Guan, Kewei Chen, Jun Zhao, Yulei Deng, Fang Xie

**Affiliations:** ^1^ Department of Nuclear Medicine & PET Center Huashan Hospital, Fudan University Shanghai China; ^2^ Department of Neurology & Institute of Neurology, Ruijin Hospital Shanghai Jiao Tong University School of Medicine Shanghai China; ^3^ Clinical Neuroscience Center Ruijin Hospital LuWan Branch Shanghai Jiao Tong University School of Medicine Shanghai China; ^4^ Department of Gerontology Shanghai Jiaotong University Affiliated Sixth People's Hospital Shanghai China; ^5^ Department of Nuclear Medicine Shanghai East Hospital Tongji University School of Medicine Shanghai China; ^6^ PET Center Department of Radiology and Biomedical Imaging Yale University School of Medicine New Haven USA; ^7^ Banner Alzheimer Institute Arizona State University, University of Arizona and Arizona Alzheimer's Consortium Phoenix USA; ^8^ State Key Laboratory of Medical Neurobiology and MOE Frontiers Center for Brain Science Fudan University Shanghai China

**Keywords:** Alzheimer's disease biomarker, *APOE* ε4, female risk, hippocampal subfields, synaptic density

## Abstract

**INTRODUCTION:**

We aimed to investigate the effect of apolipoprotein E4 (*APOE)* ε4 on synaptic density in cognitively impaired (CI) participants.

**METHODS:**

One hundred ten CI participants underwent amyloid positron emission tomography (PET) with ^18^F‐florbetapir and synaptic density PET with ^18^F‐SynVesT‐1. We evaluated the influence of *APOE* ε4 allele on synaptic density and investigated the effects of ε4 genotype on the associations of synaptic density with Alzheimer's disease (AD) biomarkers. The mediation effects of AD biomarkers on ε4‐associated synaptic density loss were analyzed.

**RESULTS:**

Compared with non‐carriers, *APOE* ε4 allele carriers exhibited significant synaptic loss in the medial temporal lobe. Amyloid beta (Aβ) and tau pathology mediated the effects of *APOE* ε4 on synaptic density to different extents. The associations between synaptic density and tau pathology were regulated by the *APOE* ε4 genotype.

**DISCUSSION:**

The *APOE* ε4 allele was associated with decreased synaptic density in CI individuals and may be driven by AD biomarkers.

## 1 BACKGROUND

The apolipoprotein E (*APOE*) ε4 allele is the strongest genetic risk factor for sporadic Alzheimer's disease (AD) and has been studied extensively. ε4 is known to be associated with abnormalities in the AD biomarkers amyloid beta (Aβ), tau pathology (T), and neurodegeneration (N), known as the “A/T/N” framework.^1^ The *APOE* ε4 allele facilitates the deposition of amyloid beta (Aβ) plaques and reduces their clearance in the brain.^2^, ^3^, ^4^ Several reports have indicated that *APOE* ε4 also increases tau pathology in AD.^5^, ^6^ Among the numerous genetic loci exhibiting sex‐specific effects on AD, the intricate interaction between sex and the *APOE* allele has been investigated in detail.^7^, ^8^ However, only a limited number of multiomics studies have been conducted to elucidate the interplay between *APOE* genotype and sex in AD.^8^ Among cognitively impaired (CI) individuals, females are more susceptible to *APOE* ε4‐associated accumulation of neurofibrillary tangles than males.^9^ In opposition to prevailing beliefs, males and females with the *APOE* ε3/ε4 genotype exhibit nearly equivalent likelihoods of developing AD between the ages of 55 and 85 years, but females exhibit a heightened risk at younger ages.^7^ Furthermore, decreased hippocampal volume and glucose uptake are both related to *APOE* ε4.^10^, ^11^

However, among these extensively reported findings, the association between the *APOE* ε4 allele and synaptic function has not been well reported or understood in humans, although this association has been reported in some animal studies, as reviewed in what follows. Synapses have been reported to play a central role in cognitive performance in AD patients. Damage or loss of synapses is significantly related to neurodegeneration. Therefore, synaptic density is considered a key biomarker of neurodegeneration.^12^ *APOE* ε4 has been proposed to aggravate synaptic dysfunction and neuronal loss. However, this phenomenon has been verified only in animal models and cerebrospinal fluid biomarkers. Mechanistically, *APOE* ε4 is considered to mediate synaptic dysfunction by interfering with Reelin signaling, which is thought to be a modulator of synaptic strength.^13^ *APOE* ε4 is also associated with synaptic damage to the cerebrospinal fluid (CSF) at the neurogranin level as well as postsynaptic density protein 95 (PSD95) and synapsin 1 (Syn1) in post mortem human brain tissue.^14^, ^15^ Cumulative Aβ, which is influenced by the *APOE* ε4 allele, can also be toxic to synapses. Synapse loss related to Aβ plaques may be caused by the increase in oligomeric Aβ rather than by the Aβ plaque itself.^16^, ^17^

**RESEARCH IN CONTEXT**

1. **Systematic review**: Apolipoprotein E ε4 (*APOE* ε4) is the strongest genetic risk factor for sporadic Alzheimer's disease (AD). However, the authors reviewed the literature using PubMed, and their review revealed that the relationship between *APOE‐*associated synaptic loss and cognitive impairment remains incompletely understood.
2. **Interpretation**: *APOE* ε4 carriers showed a significantly decreased synaptic density compared to non‐carriers, and only one copy of *APOE* ε4 reduced the synaptic density. This effect was partially mediated by amyloid pathology and fully mediated by tau pathology. *APOE* ε4 also potentiated the association between synaptic density and tau pathology.
3. **Future directions**: This study emphasized the importance of developing genotype‐guided therapies targeting synapses and related protein aggregates in AD. Large sample size and longitudinal data are needed to validate the effect of *APOE* ε4 on synaptic density loss.

Synaptic vesicle glycoprotein 2A (SV2A) is ubiquitously expressed at synapses in the central nervous system^18^ and is specifically localized within synaptic vesicles situated at presynaptic terminals.^19^ It is a direct biomarker of synaptic density in vivo.^20^ The development of SV2A radioligands, such as ^11^C‐UCB‐J and ^18^F‐SynVesT‐1, for positron emission tomography (PET) has provided an opportunity to evaluate synaptic density in the brain in various neurodegenerative disorders. Reduced synaptic density was observed across the hippocampus and neocortex in AD patients by SV2A PET.^21^ The hippocampus is considered one of the earliest affected brain regions in AD, and dysfunction of this region is believed to be the core feature of disease‐related memory impairment; moreover, analysis of this subfield could enhance the predictive value of the hippocampus in AD.^22^ Furthermore, hippocampal synaptic density has also been assessed for its association with the “A/T/N” biomarkers in AD. Hippocampal synaptic density has a significant correlation with global amyloid deposition in participants with amnestic mild cognitive impairments.^23^ Regional synaptic loss follows tau accumulation after 2 years, which indicates that tau spread might drive synaptic vulnerability.^24^

As mentioned earlier, several investigations have been performed to explain the influence of the *APOE* ε4 allele on synaptic density. However, direct evidence about the effect of *APOE* ε4 on brain synaptic loss is lacking. In this study, we examined the effect of *APOE* ε4 on synaptic density in individuals with cognitive impairment by using SV2A PET and tested whether *APOE* ε4 has diverse effects on subfields of the hippocampus. Furthermore, we investigated the effect of *APOE* ε4 on the associations between synaptic density and AD biomarkers. We hypothesized that *APOE* ε4 exerts a significant influence on synaptic density throughout the brain, which could be mediated by AD pathology. In addition, we expected a potential interaction between *APOE* ε4 genotype and sex.

## 2 METHODS

### 2.1 Participants

All participants were aged between 50 and 80 years and were recruited from memory clinics in Shanghai. The exclusion criteria are detailed in the supplementary information. All participants completed comprehensive neuropsychological assessments (described in the supplementary methods), *APOE* genotyping, ^18^F‐florbetapir PET/CT (Biograph128.mCT, Siemens, Germany), and ^18^F‐SynVesT‐1 PET/MR (uPMR790 TOF, United Imaging, China). Seventy‐four participants underwent ^18^F‐MK6240 PET/CT (Biograph128.mCT; Siemens, Germany) to survey tau pathology in the brain. Assessment of the amyloid PET images was performed through visual inspection by three experienced raters by consensus.^25^ All tests and image acquisition were conducted over 1 month.

Patients diagnoses were determined by consensus diagnostic meetings with multiple specialists. The CI participants included individuals with AD dementia and those with mild cognitive impairment (MCI). AD was diagnosed according to the 2011 National Institute on Aging and Alzheimer's Association (NIA‐AA) diagnostic criteria after comprehensive neuropsychological assessments.^26^ Individuals with MCI were identified using the approach proposed by Jak and Bondi, as described in our previous report.^27^ Cognitively unimpaired participants were defined as those who had no cognitive impairment, specifically those who did not meet the diagnostic criteria for AD or MCI, and were excluded from this study. This study was approved by the Institutional Ethics Review Board of Huashan Hospital. Written informed consent was obtained from all participants.

### 2.2 PET data acquisition and processing

Participants underwent 30‐min ^18^F‐SynVesT‐1 PET scans at 60 min after injection (6.5 ± 0.65 mCi ^18^F‐SynVesT‐1) with a 3T uPMR790 TOF (United Imaging Healthcare, China). T1‐weighted images were collected at the same time (repetition time [TR] = 7.2 ms; echo time [TE] = 3.0 ms). For ^18^F‐florbetapir PET/CT scans, 10 mCi (± 10%) [^18^F]‐florbetapir was injected, and a 20‐min scan was performed 50 min after the injection. For ^18^F‐MK6240 PET/CT scans, 5 mCi (± 10%) [^18^F]‐MK6240 was injected, and a 20‐min scan was performed 90 min after the injection. PET images were reconstructed by the filtered back projection (FBP) algorithm. For each PET imaging tracer, there was one time frame consisting of 30 and 20 min, depending on the tracer used. ^18^F‐SynVesT‐1 PET attenuation correction was performed using a three‐compartment model (bone, soft tissue, air) attenuation map automatically generated from an ultrashort TE MRI sequence. A low‐dose CT scan was used to perform ^18^F‐florbetapir and ^18^F‐MK6240 PET attenuation correction.

SPM12 was used to process the ^18^F‐florbetapir, ^18^F‐MK6240, and ^18^F‐SynVesT‐1 PET images. The detailed preprocessing steps for the voxelwise and region of interest (ROI) analyses are listed in the supplementary information. The cerebellum crus was used as a reference area to calculate the voxelwise standardized uptake value ratio (SUVR) of ^18^F‐florbetapir.^28^ Global SUVR values for ^18^F‐florbetapir were calculated by weighted averaging of the posterior cingulate, precuneus, and temporal, frontal, and parietal lobes, as described in our previous study.^29^ The whole cerebellum and inferior cerebellum were used as reference areas to calculate the voxelwise SUVRs of ^18^F‐SynVesT‐1 and ^18^F‐MK6240, respectively.^30^, ^31^, ^32^, ^33^ We chose the ROI for ^18^F‐SynVesT‐1 PET images in this study according to the findings of a previous study; the ROI encompassed the medial temporal lobe, entorhinal region, hippocampus, and parahippocampal gyrus.^34^ The masks of the hippocampal subfields were created by FreeSurfer (Laboratory for Computational Neuroimaging version 6.0, Boston, MA, USA).^35^ The hippocampal subfields were further defined as the hippocampal head, hippocampal body, and hippocampal tail, as shown in the supplementary materials (Figure S1). The hippocampal gray matter volume was calculated by CAT12.

### 2.3 Statistical analysis

Group comparisons between ε4 carriers and ε4 non‐carriers were performed using Fisher's exact test for categorical variables and the two‐sample *T* test for continuous variables such as scores on neuropsychological assessments, age, and education years. The voxelwise and ROI‐wise differences in synaptic density among ε4 carriers and ε4 non‐carriers were analyzed by a general linear model (GLM) using SPM12 and SPSS (IBM version 26.0), respectively. Age, sex, and educational level were set as covariates, and a value of *p* < 0.001 was set as the significance threshold for the voxelwise analysis. Voxelwise analyses were repeated using partial volume‐corrected data.

For ROI‐wise analysis, association coefficients were calculated by partial correlation after adjusting for age, sex, and educational level. Fisher's Z test was used to compare coefficients between ε4 carriers and ε4 non‐carriers. Statistical significance was defined as an unadjusted two‐sided *p* value less than 0.05. Mediation effects were assessed using the SPSS PROCESS macro (version 3.3). Interaction effects were calculated with the R package stargazer (version 5.2.3).

## 3 RESULTS

### 3.1 Demographics

In total, 110 participants, including 63 AD participants and 47 MCI participants, were enrolled in this study. Among the participants, 64 participants were *APOE* ε4 carriers (54 heterozygotes and 10 homozygotes) and 46 were *APOE* ε4 non‐carriers (seven individuals with the ε2ε3 genotype and 39 individuals with the ε3ε3 genotype). As shown in Table 1, compared to *APOE* ε4 non‐carriers, *APOE* ε4 carriers had a greater frequency of Aβ PET positivity and more global Aβ deposition (51/64 [79.7%] vs 24/46 [52.2%], *p* = 0.002; global Aβ SUVR: 1.45 ± 0.21 vs 1.38 ± 0.18, *p* = 0.015). *APOE* ε4 carriers were younger than *APOE* ε4 non‐carriers (66.00 ± 8.63 vs 69.63 ± 7.76, *p* = 0.025). There were no differences in sex ratio, education level, or neuropsychological scores between the *APOE* ε4 carriers and non‐carriers. Furthermore, there were no differences between the *APOE* ε4 non‐carriers and carriers among female and male patients separately, except that *APOE* ε4 carriers had a higher Aβ PET‐positive rate than non‐carriers in the female group (Table S1, 82.5% vs 40.0%, *p* < 0.001). Compared to participants in the MCI group, the participants in the AD group had greater amyloid deposition, lower education levels, and worse cognitive performance (Table S2).

**TABLE 1:** Demographic and neuropsychological testing data for participants.

Characteristic	*APOE* ε4 non‐carriers (*n* = 46)	*APOE* ε4 carriers (*n* = 64)	ε4 carriers versus ε4 non‐carriers
Sex, male: female ratio (% female)	21/25 (54.3%)	24/40 (62.5%)	*p* = 0.391
Diagnosis, AD: MCI (% AD)	24/22 (52.2%)	39/25 (60.9%)	*p* = 0.359
Age (years, mean ± SD)	69.63 ± 7.76	66.00 ± 8.63	*p* = 0.025
Educational level (years, mean ± SD)	10.82 ± 3.74	10.83 ± 3.37	*p* = 0.985
*APOE* genotype			
ε2/ε3	7	0	
ε3/ε3	39	0	
ε3/ε4	0	54	
ε4/ε4	0	10	
MMSE score (mean ± SD)	21.91 ± 6.52	21.11 ± 6.15	*p* = 0.511
MoCA‐B score (mean ± SD)	17.20 ± 7.37	15.89 ± 7.34	*p* = 0.360
AVLT‐N5 score (mean ± SD)	1.17 ± 1.58	0.72 ± 1.60	*p* = 0.197
AVLT‐N7 score (mean ± SD)	15.64 ± 4.07	14.75 ± 4.00	*p* = 0.303
BNT score (mean ± SD)	19.72 ± 5.89	17.68 ± 6.33	*p* = 0.128
AFT score (mean ± SD)	8.40 ± 3.96	7.68 ± 4.14	*p* = 0.376
STT‐A score (mean ± SD)	117.36 ± 160.03	149.82 ± 173.69	*p* = 0.371
STT‐B score (mean ± SD)	311.36 ± 291.70	437.27 ± 362.97	*p* = 0.070
Global amyloid beta (Aβ) deposition (SUVr ± SD)	1.38 ± 0.18	1.45 ± 0.21	*p* = 0.015
Aβ PET positive rate	24/46 (52.2%)	51/64 (79.7%)	*p* = 0.002

Abbreviations: AFT, Animal Fluency Test; AVLT, Auditory Verbal Learning Test; BNT, Boston Naming Test; MMSE, Mini‐Mental State Examination; MoCA‐B, Montreal Cognitive Assessment‐Basic; STT, Shape Trail Test.

### 3.2 Effect of *APOE* on synaptic density

**FIGURE 1:**
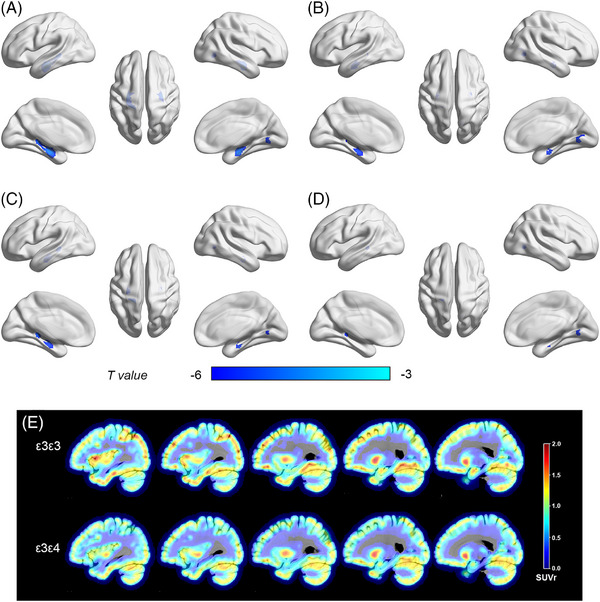
Impact of *APOE* ε4 on synaptic density loss. Voxelwise analyses (A–D) corrected to *p* < 0.001. (A) ε4 non‐carriers versus ε4 carriers without controlling for global cortical amyloid deposition and (B) controlling for global cortical amyloid deposition; (C) ε3ε3 versus ε3ε4 without controlling for global cortical amyloid deposition and (D) controlling for global cortical amyloid deposition. (E) ε3/ε3 and ε3/ε4 participants.

We first investigated the effects of *APOE* ε4 on regional synaptic density. The *APOE* ε4 carriers displayed more severe synaptic loss across the medial temporal and occipital cortices than the *APOE* ε4 non‐carriers (Figure 1A). This trend was retained in the medial temporal and occipital cortices after adjusting for the global florbetapir SUVR (Figure 1B). Furthermore, compared with those with the ε3ε3 genotype, the individuals with the ε3ε4 genotype also exhibited significant synaptic density loss in the medial temporal and occipital cortices (Figure 1C). These differences were retained after adjusting for the global florbetapir SUVR (Figure 1D). The analysis conducted with partial volume correction (PVC) (Figure S2A‐D) yielded similar results as those obtained with non‐PVC.

**FIGURE 2:**
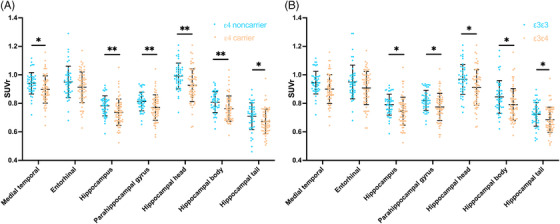
Impact of *APOE* ε4 on synaptic density loss based on ROI‐wise analysis. (A) ε4 non‐carriers versus ε4 carriers and (B) ε3ε3 versus ε3ε4. *P* values adjusted for age, education, sex, amyloid β global deposition (Aβ), and clinical diagnosis (diagnosis) are listed in Table 2. *p < 0.05, **p < 0.01.

Moreover, we observed an *APOE* ε4 effect on synaptic loss in the medial temporal (0.90 ± 0.10 vs 0.94 ± 0.07, *p* = 0.012), hippocampus (0.74 ± 0.09 vs 0.78 ± 0.07, *p* = 0.004), parahippocampal gyrus (0.77 ± 0.09 vs 0.81 ± 0.07, *p* = 0.003), hippocampal head (0.93 ± 0.12 vs 0.99 ± 0.09, *p* = 0.001), hippocampal body (0.76 ± 0.09 vs 0.81 ± 0.07, *p* = 0.003), and hippocampal tail (0.67 ± 0.09 vs 0.71 ± 0.10, *p* = 0.011) without controlling for global amyloid burden. After controlling for the amyloid burden, the effect was decreased but still significant in the majority of regions such as in the hippocampus (*p* = 0.004 vs 0.040), parahippocampal gyrus (*p* = 0.003 vs 0.031), hippocampal head (*p* = 0.001 vs 0.014), and hippocampal body (*p* = 0.003 vs 0.045) (Figure 2A, Table 2).

**TABLE 2:** *P* value adjusted for age, education, sex, amyloid beta (Aβ) global deposition, and clinical diagnosis (diagnosis).

Groups compared	Regions of interest	*p* value adjusting for age, education, sex	*p* value adjusting for age, education, sex, Aβ	*p* value adjusting for age, education, sex, Aβ, diagnosis
ε4 non‐carriers versus ε4 carriers	Medial temporal lobe	0.012	0.073	0.075
	Entorhinal	0.093	0.284	0.291
	Hippocampus	0.004	0.040	0.041
	Parahippocampal gyrus	0.003	0.031	0.032
	Hippocampal head	0.001	0.014	0.014
	Hippocampal body	0.003	0.045	0.046
	Hippocampal tail	0.011	0.081	0.081
ε3ε3 participants versus ε3ε4 participants	Medial temporal lobe	0.058	0.197	0.214
	Entorhinal	0.298	0.661	0.772
	Hippocampus	0.021	0.093	0.100
	Parahippocampal gyrus	0.016	0.071	0.079
	Hippocampal head	0.014	0.059	0.066
	Hippocampal body	0.021	0.101	0.106
	Hippocampal tail	0.013	0.051	0.051

The *APOE* ε4 effect on synaptic loss was also observed between the individuals with the ε3ε4 genotype and individuals with the ε3ε3 genotype (Figure 2B, Table 2) in the hippocampus (0.74 ± 0.10 vs 0.78 ± 0.07, *p* = 0.021), parahippocampal gyrus (0.77 ± 0.09 vs 0.82 ± 0.07, *p* = 0.016), hippocampal head (0.93 ± 0.12 vs 0.99 ± 0.10, *p* = 0.014), hippocampal body (0.77 ± 0.09 vs 0.81 ± 0.07, *p* = 0.021), and hippocampal tail (0.68 ± 0.09 vs 0.72 ± 0.09, *p* = 0.013) without controlling for amyloid. After controlling for the amyloid burden, the effect was also decreased, such as in the hippocampus (*p* = 0.021 vs .093), parahippocampal gyrus (*p* = 0.016 vs 0.071), hippocampal head (*p* = 0.014 vs 0.059), hippocampal body (*p* = 0.021 vs 0.101), hippocampal tail (*p* = 0.013 vs 0.051).

### 3.3 Effect on synaptic density loss in different sex

We observed significant main effects of *APOE* ε4 on synaptic density based on the ROIs, although no interaction effect was found in any of the ROIs or voxels (Table S3). Sex‐stratified voxelwise analysis revealed that, compared with female *APOE* ε4 non‐carriers, female *APOE* ε4 carriers had decreased synaptic density, mainly in the bilateral hippocampus, with both non‐PVC and PVC (Figure S3A‐D). Specifically, ROI‐wise analysis revealed that female *APOE* ε4 carriers exhibited significant synaptic density loss in the hippocampus, parahippocampal gyrus, and hippocampal head, body, and tail (Figure S3G). Compared to male *APOE* ε4 non‐carriers, male *APOE* ε4 carriers showed a loss of synaptic density in the medial temporal regions according to voxelwise analyses (Figure S3E) and in the hippocampal head (Figure S3H) without controlling for the global florbetapir SUVr.

### 3.4 *APOE*‐stratified effect of association between synaptic density and “A/T/N” biomarkers

We used partial correlations to investigate the association between synaptic density and “A/T/N” biomarkers while controlling for sex, age, and education level. First, in the overall cohort, we found that hippocampal synaptic density was associated with global amyloid deposition (*R* = −0.446, *p* < 0.001), medial temporal tau deposition (*R* = −0.568, *p* < 0.001), and hippocampal volume (*R* = 0.699, *p* < 0.001). Synaptic density in the parahippocampal gyrus was also associated with global amyloid deposition (*R* = −0.420, *p* < 0.001), medial temporal tau deposition (*R* = −0.554, *p* < 0.001), and hippocampal volume (*R* = 0.649, *p* < 0.001).

**FIGURE 3:**
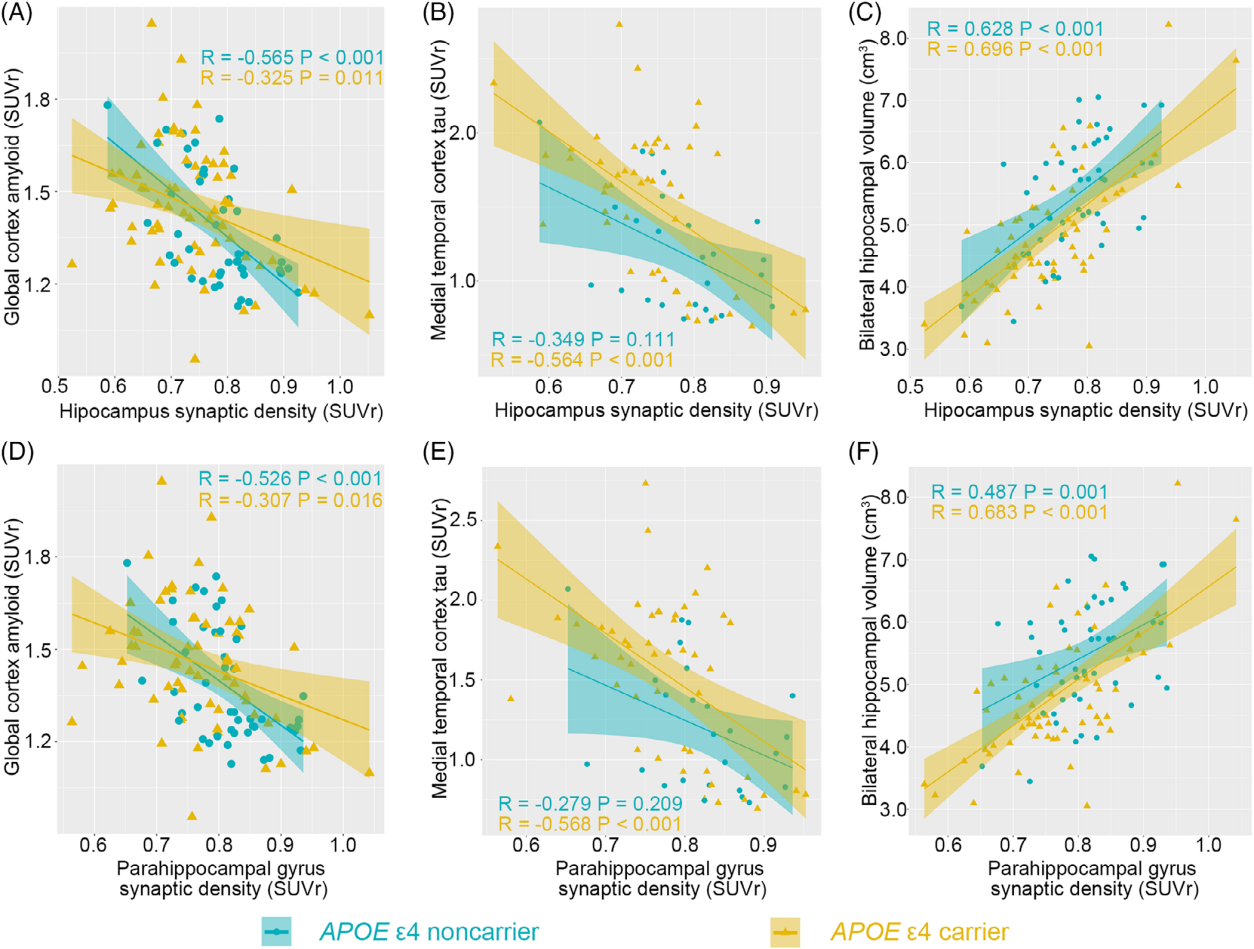
*APOE* genotype modifies the association between synaptic density and “A/T/N” biomarkers. *APOE* genotype modified the association of synaptic density in the hippocampus and parahippocampal gyrus with (A, D) global amyloid deposition, (B, E) medial temporal tau deposition, and (C, F) bilateral hippocampal volume.

According to our stratified analysis, hippocampal synaptic density was negatively associated with global amyloid deposition in *APOE* ε4 carriers (*R* = −0.325, *p* = 0.011) and *APOE* ε4 non‐carriers (*R* = −0.565, *p* < 0.001; Figure 3A). In addition, the synaptic density in the parahippocampal gyrus was associated with global amyloid deposition in *APOE* ε4 non‐carriers (*R* = −0.526, *p* < 0.001) and *APOE* ε4 carriers (*R* = −0.307, *p* = 0.016; Figure 3D). In a subgroup of participants who underwent ^18^F‐MK6240, we found that medial temporal tau deposition was associated with synaptic density in the hippocampus and parahippocampal gyrus in *APOE* ε4 carriers (*R* = −0.564, *p* < 0.001; Figure 3B; *R* = −0.568, *p* < 0.001; Figure 3E) but not in *APOE* ε4 non‐carriers (*R* = −0.349, *p* = 0.111; Figure 3B; *R* = −0.279, *p* = 0.209; Figure 3E). Bilateral hippocampal volume was associated with synaptic density in the hippocampus and parahippocampal gyrus in both *APOE* ε4 carriers (*R* = 0.696, *p* < 0.001; Figure 3C; *R* = 0.683, *p* < 0.001; Figure 3F) and *APOE* ε4 non‐carriers (*R* = 0.628, *p* < 0.001; Figure 3C; *R* = 0.487, *p* = 0.001; Figure 3F).

### 3.5 Synaptic density loss mediated by Aβ and tau pathology

**FIGURE 4:**
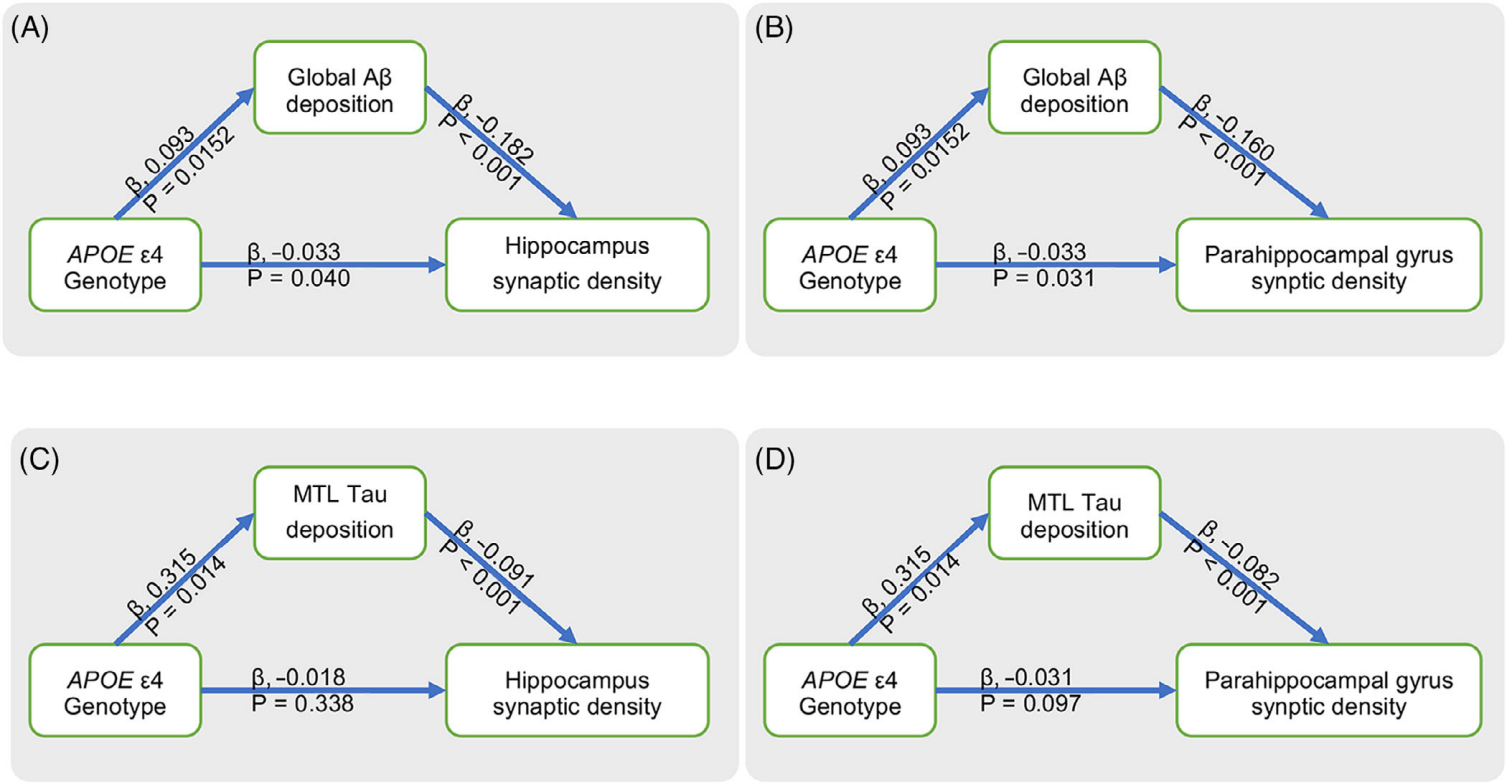
Mediation effects of Aβ/tau pathology on relationship between *APOE* ε4 genotype and synaptic density. (A, B) Mediating effects of global Aβ PET SUVr on association between *APOE* ε4 genotype and synaptic density in hippocampus (indirect effects = −0.017, SE = 0.008, 95% CI = −0.036 to −0.003; direct effects = −0.033, SE = 0.016, 95% CI = −0.066 to −0.002) and parahippocampal gyrus (indirect effects = −0.015, SE = 0.007; 95% CI = −0.031 to −0.003; direct effects = −0.033, SE = 0.015, 95% CI = −0.063 to −0.003). (C, D) Mediating effects of medial temporal cortex tau PET SUVr on relationship between *APOE* ε4 genotype and synaptic density in hippocampus (indirect effects = −0.029, SE = 0.011, 95% CI = −0.052 to −0.008; direct effects = −0.018, SE = 0.019, 95% CI = −0.056 to 0.020) and parahippocampal gyrus (indirect effects = −0.026, SE = 0.010, 95% CI = −0.046 to −0.007; direct effects = −0.031, SE = 0.018, 95% CI = −0.067 to 0.006).

We further performed a mediation analysis to investigate the correlation among *APOE* ε4 genotype, Aβ (or tau) pathology, and synaptic density in the hippocampus and parahippocampal gyrus. We first assessed whether a higher *APOE* ε4‐conferred Aβ burden was associated with severe synaptic density loss. Bootstrapped mediation analyses revealed that the association between the *APOE* ε4 genotype and synaptic density in the hippocampus or parahippocampal gyrus was partially mediated by the global Aβ PET SUVR (Figure 4A and B, hippocampus: indirect effects = −0.017, SE = 0.008, 95% CI = −0.036 to −0.003; direct effects = −0.033, SE = 0.016, 95% CI = −0.066 to −0.002; parahippocampal gyrus: indirect effects = −0.015, SE = 0.007; 95% CI = −0.031 to −0.003; direct effects = −0.033, SE = 0.015, 95% CI = −0.063 to −0.003). Second, we tested whether the *APOE* ε4 genotype accelerated decreased synaptic density via increased medial temporal cortex tau PET SUVR accumulation. The relationship between the *APOE* ε4 genotype and synaptic density in the hippocampus (Figure 4C, indirect effects = −0.029, SE = 0.011, 95% CI = −0.052 to −0.008; direct effects = −0.018, SE = 0.019, 95% CI = −0.056 to 0.020) and the parahippocampal gyrus (Figure 4D, indirect effects = −0.026, SE = 0.010, 95% CI = −0.046 to −0.007; direct effects = −0.031, SE = 0.018, 95% CI = −0.067 to 0.006) was fully mediated by the medial temporal tau PET SUVR.

## 4 DISCUSSION

The *APOE* ε4 allele was determined to exert different effects on AD biomarkers, such as amyloid and tau deposition. However, the mechanism underlying synaptic loss in *APOE* ε4 carriers remains unclear. Although various studies have shown that *APOE* ε4 is associated with impaired synaptic integrity, these observations have not been validated in living human brains. In this study, we found that *APOE* ε4 was associated with a loss of synaptic density in CI participants. Only one copy of *APOE* ε4 is sufficient to induce synaptic density loss. However, we did not observe a significant sex–*APOE* interaction effect on synaptic density. Aβ pathology partially mediated the relationship between the *APOE* ε4 genotype and synaptic density. However, tau pathology fully mediated the relationship between the *APOE* ε4 genotype and synaptic density.

In this study, we confirmed that *APOE* ε4 carriers might undergo synaptic damage, which confers additional risk of AD. First, we found that *APOE* ε4 carriers exhibited significantly reduced synaptic density in the medial temporal lobe and neocortex. The reduced synaptic density remained after controlling for florbetapir SUVR, which may partly explain the synaptic loss associated with the *APOE* ε4 allele. Some data demonstrate that the soluble amyloid protein is toxic to synapses.^36^, ^37^ Although evidence indicates that the *APOE* genotype is associated with synaptic function in animal models, investigations regarding the effect of *APOE* on synaptic function in humans are limited. To our knowledge, this is the first report of significantly reduced synaptic density in the living human brain.

Previous studies also revealed a significant decrease in synaptic density in the medial temporal lobe and neocortex in patients with AD compared to that in healthy controls. We found that this reduction focused on the medial temporal lobe in *APOE* ε4 carriers compared to that in *APOE* ε4 non‐carriers. Synaptic density reduction in the hippocampus is considered the main pathology of AD. Lipidated apoE also regulates neuronal maintenance and repair by regenerating nerve cells to facilitate the repair process after neuronal injury.^38^ ApoE4 expressed by neuron could resulted in loss of neurons.^39^ Therefore, *APOE* ε4 could accelerate synaptic impairment, especially hippocampal synaptic impairment. Pathological analysis revealed that in the early stage of AD, the hippocampus experiences a rapid loss of tissue, which is correlated with the accumulation of amyloid plaques and tau tangles.^40^ Specifically, a reduction in synaptic density in the hippocampal head was significant. A previous tau PET study demonstrated that the earliest tau accumulation occurs in the entorhinal cortex, Brodmann area 35, and anterior hippocampus and could be associated with the loss of synapse density in the hippocampal head.^41^ These studies suggested that segmenting the lateral hippocampus into three subregions (head, body, and tail) could be useful for understanding the progressive pathological changes in the hippocampus in AD patients. However, additional studies are needed to elucidate the underlying mechanism.

The *APOE* genotype also regulates the association between synaptic density and AD biomarkers. Global amyloid deposition has been associated with hippocampal synaptic density.^42^ *APOE* ε4 potentiated the association between synaptic density and tau pathology. apoE immunoreactivity demonstrated that increased expression of apoE in neurons is associated with increased tau phosphorylation.^43^ Tau pathology is closely related to neurodegeneration and cognitive performance. *APOE* ε4 promotes increased tau deposition and brain atrophy in carriers compared to non‐carriers, which is proposed to be mediated by glia.^44^ Astrocytes are the main source of apoE, and signaling by astrocytes results in synaptic and glial transmission.^1^ Therefore, we observed a close association between synaptic density and tau deposition and hippocampal volume. In addition, analysis of the mediation model suggested that the effects of *APOE* ε4 on synaptic loss were not independent and could be driven by amyloid/tau pathology. Amyloid pathology partially mediated the effects of *APOE* ε4 on synaptic loss and may be correlated with increasing oligomeric Aβ levels rather than Aβ plaques. Importantly, tau pathology in the medial temporal lobe fully mediated the effects of *APOE* ε4 on synaptic loss, which supports the hypothesis that synapses are damaged due to the toxicity of tau.^45^, ^46^

This study has several limitations. First, we did not have longitudinal data to validate the effect of *APOE* ε4 on synaptic density loss. Second, due to the limited resolution of PET, the analysis power of subfields of the hippocampus was limited. Finally, additional studies of cognitively unimpaired patients are needed to investigate the effect of *APOE* ε4 on synaptic density loss in these individuals.

In this study, we found that *APOE* ε4 played a significant role in synaptic loss. Compared with non‐carriers, *APOE* ε4 carriers exhibited significantly decreased synaptic density in the medial temporal lobe, and only one copy of *APOE* ε4 was sufficient to reduce the synaptic density in the hippocampus. This effect was partially mediated by amyloid pathology and fully mediated by tau pathology. *APOE* ε4 also potentiated the association between synaptic density and tau pathology. This study emphasized the importance of developing genotype‐guided therapies targeting synapses and related protein aggregates in AD.

## AUTHOR CONTRIBUTIONS

Fang Xie, Yulei Deng, Binyin Li, and Jun Zhao designed this study and coordinated all the research. Fang Xie, Yiyun Henry Huang, and Qihao Guo organized the data collection. Kun He, Jie Wang, Ying Wang, Zhiwen You, Xing Chen, Haijuan Chen, and Junpeng Li collected the data. Kun He and Jie Wang processed and analyzed the data. Yiyun Henry Huang reviewed the study design and revised the study. Fang Xie led the writing of the manuscript, and all the authors reviewed and revised the manuscript.

## CONFLICT OF INTEREST STATEMENT

FX is a consultant for Zentera Therapeutics (Shanghai) Co., Ltd. The other authors declare no conflicts of interest. Author disclosures are available in the supporting information.

## ROLE OF FUNDERS/SPONSORS

The funders had no role in the design or conduct of the study; collection, management, analysis, or interpretation of the data; preparation, review, or approval of the manuscript; or decision to submit the manuscript for publication.

## CONSENT FOR PUBLICATION

Not applicable.

## CONSENT STATEMENT

Written informed consent was obtained from all participants.

## Supporting information

Supporting information

Supporting information
