# Supplementary material for: APOE ε4 is associated with decreased synaptic density in cognitively impaired participants
Source: Alzheimers Dement. 2024 Mar 13;20(5):3157–66. doi: 10.1002/alz.13775 (PMC11095422; doi:10.1002/alz.13775)
Supplement: Supplementary file 1 — Supporting information [file ALZ-20-3157-s001.docx]

**Supplementary material**

**Supplementary methods**

### Participants exclusion criteria

Patients with neurological or psychiatric antecedents, severe alcohol or drug abuse, disorders of the central nervous system, and serious diseases such as cancers were excluded. Participants with any magnetic resonance imaging (MRI) or PET contraindications were also excluded.

### Neuropsychological assessments

All participants were given comprehensive neuropsychological assessments [1, 2]. Two global cognition tests and three cognitive domains containing six neuropsychological tests were conducted. Global cognition was assessed using the Mini-Mental State Examination (MMSE) and Montreal Cognitive Assessment-Basic (MoCA-B); memory function was assessed using long-delayed recall of the auditory verbal learning test (AVLT-LDR) and AVLT recognition; language function was assessed using the animal fluency test (AFT, total score) and 30-item Boston naming test (BNT, total score); and executive function was assessed using shape trail test (STT) parts A and B (time to completion) [3, 4].

### PET data processing

For voxel-wise analysis, this brief process was conducted as follows: the PET images were first coregistered to their respective T1-weighted images (TE: 3.0ms, TR: 7.19ms, Slice Thickness: 0.67mm), and then the PETPVE12 Muller-Gartner was used to accomplish voxel-wise partial volume error correction (PVC). All images were further warped into the normal MNI stereotactic space using transformational parameters from the MRI warping. Finally, a Gaussian smoothing kernel with an 8 mm full width at half maximum (FWHM) was used to smooth the images. For ROI (Regions of interest)-wise analysis, PET images were first coregistered to their respective T1-weighted images. All images were further warped into the normal MNI stereotactic space using transformational parameters from the MRI warping. Finally, a Gaussian smoothing kernel with an 8 mm full width at half maximum (FWHM) was used to smooth the images. Then, the images for ROI-wise analysis were masked using a mask based on the Automated Anatomical Labeling (AAL) atlas.

### MRI data acquisition

All scanning was performed on a 3T uPMR790 TOF (United Imaging Healthcare, China). using an eight-channel phased-array head coil. Foam padding and earplugs were used to minimize head movement and scanner noise. During scanning, participants were often reminded to remain motionless with eyes closed, without falling asleep, and without thinking of anything special (confirmed by subjects immediately after the experiment).

High-resolution T1 images were acquired by 3-D magnetization-prepared rapid gradient-echo sequence as follows: repetition time 7.19 ms, echo time 3.0 ms, flip angle 10°, trans-axial acquisition matrix = 256 × 329, in-plane resolution = 1mm × 1mm, slice thickness = 1mm, sagittal slice = 176.

**
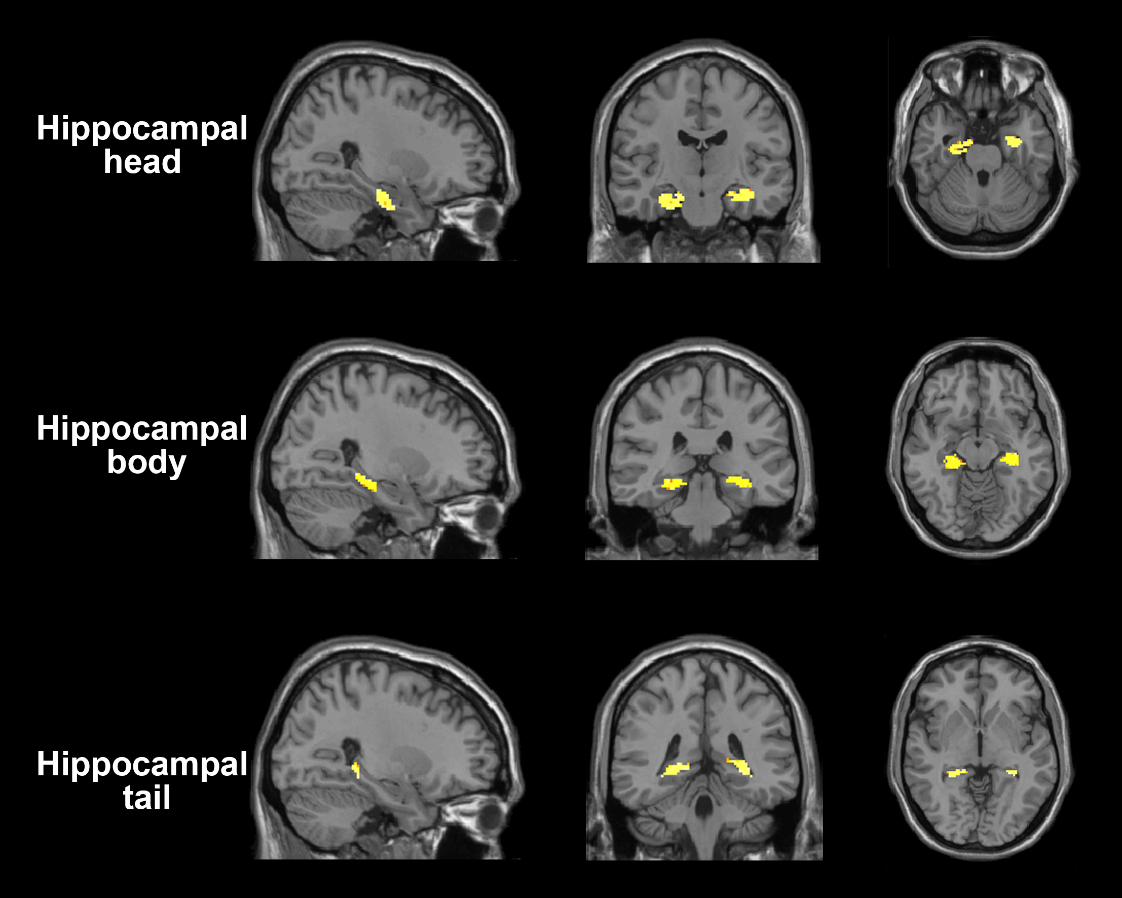
**

**Figure S1.** The hippocampal subfields in this study.

| Parasubiculum | **HEAD** |
| --- | --- |
| Presubiculum-head |  |
| Subiculum-head |  |
| CA1-head |  |
| CA2-head |  |
| CA3-head |  |
| GC-ML-DG-head |  |
| Molecular_layer_HP-head |  |
| HATA |  |
| Presubiculum-body | **BODY** |
| Subiculum-body |  |
| CA1-body |  |
| CA2-body |  |
| CA3-body |  |
| GC-ML-DG-body |  |
| Molecular_layer_HP-body |  |
| fimbria |  |
| Hippocampal_tail | **TAIL** |

**
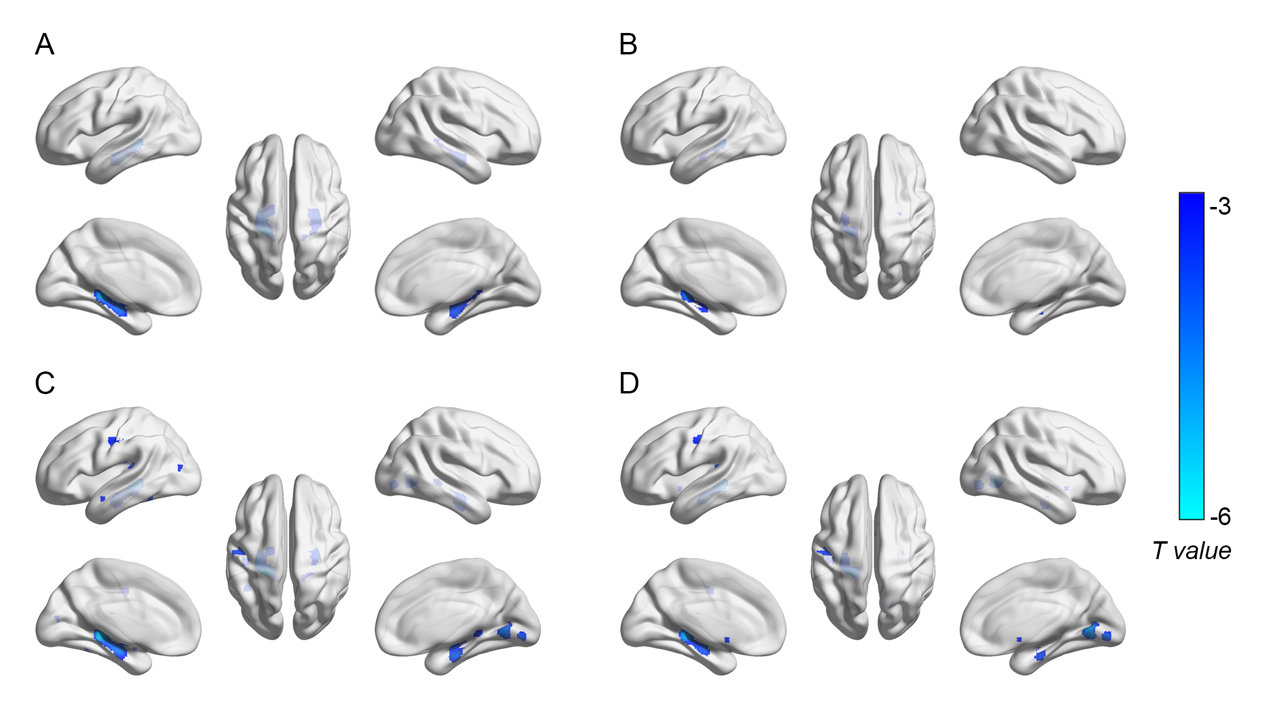
**

**Figure S2.** *APOE* ε4 effect on synaptic density loss

(A-D) had accomplished partial volume error correction (PVC) and been corrected by P < 0.001. (A) Voxel-wise analysis between ε4 noncarriers and ε4 carriers without controlling for global cortical amyloid deposition and (B) controlling for global cortical amyloid deposition; (C) Voxel-wise analysis between individuals with ε3ε3 and individuals with ε3ε4 without controlling for global cortical amyloid deposition and (D) controlling for global cortical amyloid deposition.

**
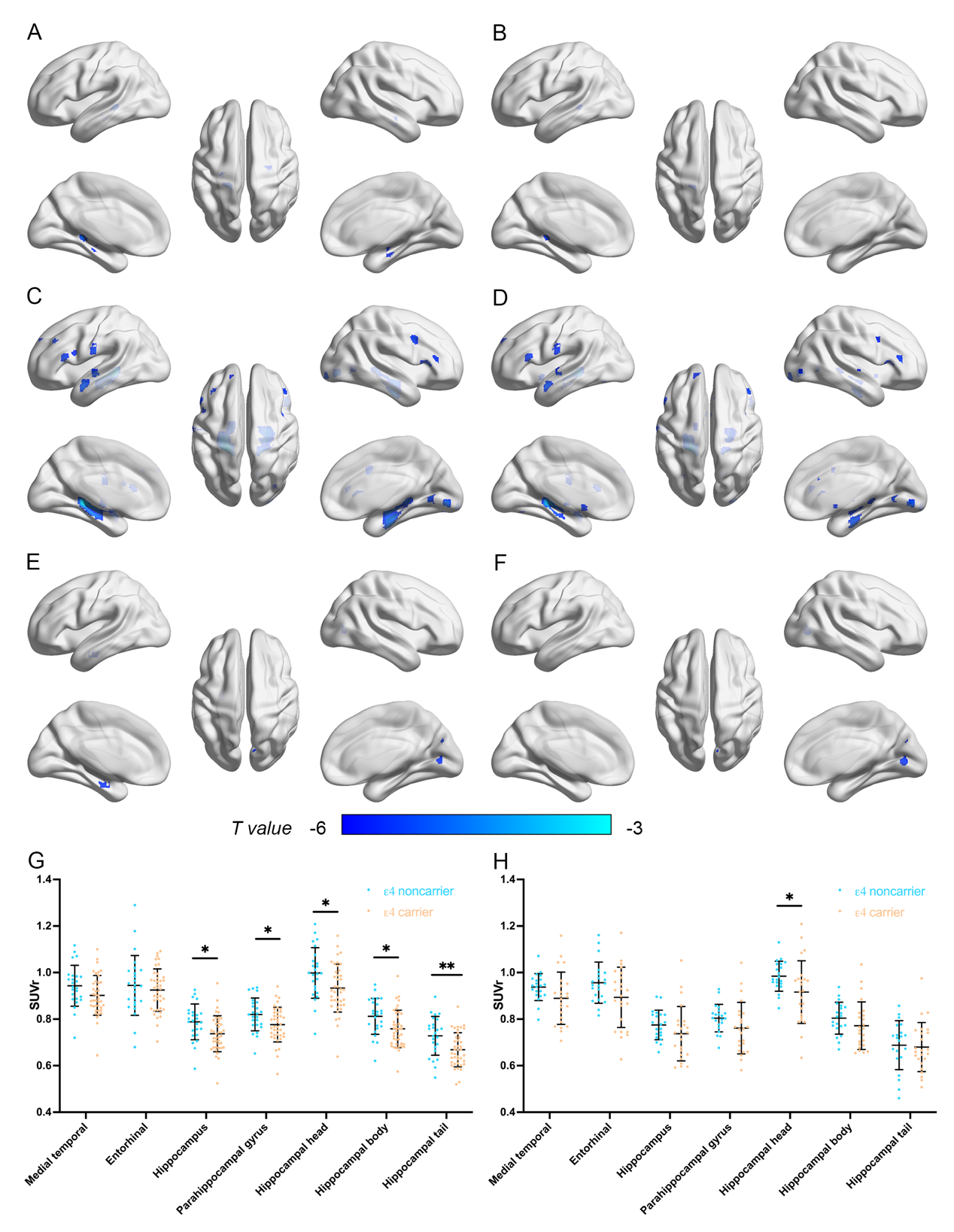
Figure S3.** *APOE* ε4 Impact on Synaptic Density Loss in Females and Males Voxel-wise analyses of Synaptic Density between ε4 noncarriers and carriers in females without (A&C) and with controlling for global cortical amyloid deposition (B&D). Voxel-wise analyses of Synaptic Density between ε4 noncarriers and carriers in males without (E) and with controlling for global cortical amyloid deposition (F). ROI-wise analyses between ε4 carriers and noncarriers in female (G) and male (H) without controlling amyloid deposition. (C&D) had accomplished partial volume error correction. *P < 0.05, **P < 0.01, ***P < 0.001

**Table S1.** Demographic and clinical characteristics by sex of the study cohort.

| **Characteristic** | **Females** | | | **Males** | | |
| --- | --- | --- | --- | --- | --- | --- |
|  | ε4 noncarriers  (n = 25) | ε4 carriers  (n = 40) | ε4 carriers vs.  ε4 noncarriers | ε4 noncarriers  (n = 21) | ε4 carriers  (n = 24) | ε4 carriers vs.  ε4 noncarriers |
| Age (years, mean ± SD) | 69.36 ± 8.23 | 65.70 ± 8.51 | 0.092 | 69.95 ± 7.35 | 66.50 ± 8.99 | 0.169 |
| Education level (years, mean ± SD) | 10.46 ± 3.48 | 10.50 ± 3.69 | 0.965 | 11.24 ± 4.07 | 11.38 ± 2.76 | 0.895 |
| ApoE genotype |  |  |  |  |  |  |
| ε2/ε3 | 2 |  |  | 5 |  |  |
| ε3/ε3 | 23 |  |  | 16 |  |  |
| ε3/ε4 |  | 33 |  |  | 21 |  |
| ε4/ε4 |  | 7 |  |  | 3 |  |
| MMSE score (mean ± SD) | 22.04 ± 5.91 | 21.05 ± 6.67 | 0.546 | 21.76 ± 7.32 | 21.21 ± 5.31 | 0.771 |
| MoCA-B score (mean ± SD) | 17.52 ± 6.08 | 16.13 ± 7.64 | 0.443 | 16.81 ± 8.80 | 15.50 ± 6.94 | 0.580 |
| AVLT-N5 score (mean ± SD) | 1.17 ± 1.54 | 1.03 ± 1.89 | 0.790 | 1.17 ± 1.65 | 0.16 ± 0.50 | 0.016 |
| AVLT-N7 score (mean ± SD) | 16.17 ± 3.37 | 14.89 ± 4.49 | 0.292 | 15.11 ± 4.71 | 14.50 ± 3.04 | 0.634 |
| AFT score (mean ± SD) | 8.30 ± 3.08 | 7.13 ± 3.59 | 0.195 | 8.50 ± 4.86 | 8.61 ± 4.88 | 0.942 |
| BNT score (mean ± SD) | 18.06 ± 4.25 | 17.35 ± 6.64 | 0.687 | 21.39 ± 6.90 | 18.26 ± 5.86 | 0.145 |
| STT-A score (mean ± SD) | 84.22 ± 48.35 | 111.44 ± 66.25 | 0.131 | 150.50 ± 219.23 | 211.95 ± 260.11 | 0.435 |
| STT-B score (mean ± SD) | 284.39 ± 268.43 | 422.29 ± 353.31 | 0.153 | 338.33 ± 318.72 | 462.24 ± 386.06 | 0.287 |
| Amyloid β global deposition (SUVr ± SD) | 1.37 ± 0.19 | 1.45 ± 0.19 | 0.079 | 1.39 ± 0.19 | 1.44 ± 0.24 | 0.412 |
| Amyloid β PET positive rate (-/+) | 15/10 (40%) | 7/33 (82.5%) | < 0.001 | 7/14 (66.7%) | 6/18 (75.0%) | 0.538 |

**Table S2.** Demographic and clinical characteristics by clinical diagnosis of the study cohort.

| **Characteristic** | **AD**  **(N = 63)** | **MCI**  **(N = 47)** | **AD vs. MCI** |
| --- | --- | --- | --- |
| Sex, male: female ratio (% female) | 26/37 (58.7%) | 19/28 (59.6%) | 0.929 |
| Age (years, mean ± SD) | 66.65 ± 8.77 | 68.68 ± 7.91 | 0.213 |
| Education level (years, mean ± SD) | 9.81 ± 3.22 | 12.18 ± 3.47 | < 0.001 |
| *APOE* genotype |  |  |  |
| ε2/ε3 | 3 | 4 |  |
| ε3/ε3 | 21 | 18 |  |
| ε3/ε4 | 33 | 21 |  |
| ε4/ε4 | 6 | 4 |  |
| *APOE* %ε4 | 61.9% | 53.2% | 0.359 |
| MMSE score (mean ± SD) | 17.43 ± 5.30 | 26.83 ± 2.06 | < 0.001 |
| MoCA-B score (mean ± SD) | 11.78 ± 5.57 | 22.68 ± 4.06 | < 0.001 |
| AVLT-N5 score (mean ± SD) | 0.12 ± 0.45 | 1.62 ± 1.91 | < 0.001 |
| AVLT-N7 score (mean ± SD) | 12.70 ± 3.65 | 17.34 ± 2.95 | < 0.001 |
| AFT score (mean ± SD) | 6.43 ± 3.40 | 9.87 ± 4.04 | < 0.001 |
| BNT score (mean ± SD) | 15.45 ± 6.32 | 21.23 ± 4.68 | < 0.001 |
| STT-A score (mean ± SD) | 203.48 ± 221.38 | 74.72 ± 37.37 | < 0.001 |
| STT-B score (mean ± SD) | 598.02 ± 364.54 | 186.91 ± 135.05 | < 0.001 |
| Amyloid β global deposition (SUVr ± SD) | 1.45 ± 0.21 | 1.38 ± 0.17 | 0.003 |
| Amyloid β PET positive rate | 12/51 (81.0%) | 23/24 (51.1%) | < 0.001 |

| **Dependent variable** | **Main Effects** | **Interaction** |
| --- | --- | --- |
| Medial temporal synaptic density | R²=0.060, Adj R²=0.043 | R²=0.061, Adj R²=0.034 |
| Sex | -0.010 (n.s.) | -0.006 (n.s.) |
| *APOE* | -0.044* | -0.041 (n.s.) |
| Sex: *APOE* | - | 0.007 (n.s.) |
|  |  |  |
| Entorhinal synaptic density | R²=0.031, Adj R²=0.013 | R²=0.040, Adj R²=0.013 |
| Sex | -0.013 (n.s.) | 0.012 (n.s.) |
| *APOE* | -0.038 (n.s.) | -0.020 (n.s.) |
| Sex: *APOE* | - | -0.043 (n.s.) |
|  |  |  |
| Hippocampus synaptic density | R²=0.067, Adj R²=0.049 | R²=0.068, Adj R²=0.042 |
| Sex | -0.006 (n.s.) | -0.013 (n.s.) |
| *APOE* | -0.045** | -0.051* |
| Sex: *APOE* | - | 0.013 (n.s.) |
|  |  |  |
| Parahippocampal gyrus synaptic density | R²=0.073, Adj R²=0.056 | R²=0.073, Adj R²=0.047 |
| Sex | -0.015 (n.s.) | -0.016 (n.s.) |
| *APOE* | -0.044** | -0.044* |
| Sex: *APOE* | - | 0.001 (n.s.) |
|  |  |  |
| Hippocampal head synaptic density | R²=0.091, Adj R²=0.074 | R²=0.091, Adj R²=0.066 |
| Sex | -0.016 (n.s.) | -0.014 (n.s.) |
| *APOE* | -0.066** | -0.065* |
| Sex: *APOE* | - | -0.003 (n.s.) |
|  |  |  |
| Hippocampal body synaptic density | R²=0.070, Adj R²=0.053 | R²=0.074, Adj R²=0.048 |
| Sex | 0.004 (n.s.) | -0.008 (n.s.) |
| *APOE* | -0.045** | -0.054* |
| Sex: *APOE* | - | 0.022 (n.s.) |
|  |  |  |
| Hippocampal tail synaptic density | R²=0.044, Adj R²=0.026 | R²=0.063, Adj R²=0.037 |
| Sex | -0.011 (n.s.) | -0.040 (n.s.) |
| *APOE* | -0.038* | -0.060* |
| Sex: *APOE* | - | 0.052 (n.s.) |

**Table S3.** Interaction effects between *APOE* and sex *p<0.05; **p<0.01; ***p<0.001; n.s.（not significant）

**References**

[1] Guo Q, Zhao Q, Chen M, Ding D, Hong Z. A comparison study of mild cognitive impairment with 3 memory tests among Chinese individuals. Alzheimer Dis Assoc Disord. 2009;23:253-9.

[2] Ding D, Zhao Q, Guo Q, Liang X, Luo J, Yu L, et al. Progression and predictors of mild cognitive impairment in Chinese elderly: A prospective follow-up in the Shanghai Aging Study. Alzheimers Dement (Amst). 2016;4:28-36.

[3] Zhao Q, Guo Q, Hong Z. Clustering and switching during a semantic verbal fluency test contribute to differential diagnosis of cognitive impairment. Neurosci Bull. 2013;29:75-82.

[4] Zhao Q, Guo Q, Liang X, Chen M, Zhou Y, Ding D, et al. Auditory Verbal Learning Test is Superior to Rey-Osterrieth Complex Figure Memory for Predicting Mild Cognitive Impairment to Alzheimer's Disease. Curr Alzheimer Res. 2015;12:520-6.
